# Kaposi’s sarcoma herpesvirus viral FLICE inhibitory protein modulates A20 deubiquitinase activity

**DOI:** 10.1099/acmi.0.000625.v4

**Published:** 2024-05-09

**Authors:** Kevin Herold, Ayana Ruffin, Jennifer C. Chmura, Anna J. Dellomo, Elana S. Ehrlich

**Affiliations:** 1 Department of Biological Sciences, Towson University, Towson, MD, USA; 2 Cancer Reserach Institute, Emory University, Atlanta, GA, USA

**Keywords:** CASP8, deubiquitinating enzymes, FADD-like apoptosis regulating protein, herpesvirus 8, human, Kaposi's sarcoma-associated herpesvirus, ubiquitin

## Abstract

KSHV viral FLICE inhibitory protein (vFLIP) is a potent activator of NF-κB signalling and an inhibitor of apoptosis and autophagy. Inhibition of vFLIP function and NF-κB signalling promotes lytic reactivation. Here we provide evidence for a novel function of vFLIP through inhibition of the deubiquitinating (DUB) activity of the negative regulator, A20. We demonstrate direct interaction of vFLIP with Itch and A20 and provide evidence for subsequent loss of A20 DUB activity. Our results provide further insight into the function of vFLIP in the regulation of NF-κB signalling.

## Data Summary

No new data, tools, software or code have been generated or are required for work to be reproduced.

## Introduction

Kaposi’s sarcoma herpesvirus (KSHV) is a member of the γ2 subfamily of herpesviruses and the causative agent of Kaposi’s sarcoma [1,2]. The KSHV genome has been found in the cells of two B-cell lymphoproliferative diseases – primary effusion lymphoma (PEL) and multicentric Castleman’s disease (MCD) – and is associated with two inflammatory syndromes, immune reconstitution inflammatory syndrome-KS (IRIS-KS) and KSHV inflammatory cytokine syndrome (KICS) [3,5]. KSHV has been classified as a group 1 carcinogen by the International Agency for Research on Cancer and the National Toxicology Program 14th Report on Carcinogens [6].

The KSHV genome contains several viral homologues of cellular genes, many of which promote immune evasion, cell survival, and proliferation. KSHV exists mostly as a latent infection, where the viral genome is tethered to the host chromosome by latency-associated nuclear antigen (LANA) and infectious virions are not produced. Nascent virions are produced during periods of lytic replication induced by expression of the viral transactivator, RTA [7].

KSHV oncogenesis is, in part, attributed to genes expressed during latency. Viral FLICE inhibitory protein (vFLIP or K13) is a latently expressed gene that was originally identified as an inhibitor of apoptosis, due to the presence of tandem death effector domains [8,9]. vFLIP is a potent activator of NF-κB signalling and this activity is dependent on interaction with IKKγ [10,12]. vFLIP has also been shown to promote NF-κB signalling through upregulation of IKKε and CADM1 and inhibition of the SAP18/HDAC1 complex, resulting in activation of NF-κB via acetylation of p65 [13,15]. NF-κB signalling is required for the virus to maintain latency, as chemical inhibition of this signalling pathway has been shown to promote lytic replication [16,17].

vFLIP also plays a role in oncogenesis and genome instability. A transgenic mouse model of vFLIP expression displays persistent NF-κB activation and an increased incidence of lymphoma as well as B-cell abnormalities similar to those observed in MCD (reviewed in [18]). More recently, vFLIP was shown to increase LINE-1 retrotransposition, which may promote genome instability [19].

NF-κB signalling induces expression of negative regulators that limit the inflammatory response. A20 (TNFAIP3), one such negative regulator of NF-κB, is induced by vFLIP. A20 is a ubiquitin-editing protein with both C-terminal ubiquitin ligase activity and N-terminal deubiquitinase (DUB) activity. In one well characterized mechanism, A20 forms a ubiquitin editing complex with Itch, RNF11, and TAX1BP1, and downmodulates NF-κB signalling through removal of K63-linked polyubiquitin chains from RIPK1 followed by addition of K48-linked polyubiquitin chains, resulting in degradation of RIPK1 via the proteasome [20]. A20 is reported to deubiquitinate a number of signalling intermediates within the NF-κB pathway in addition to RIPK1, including IKKγ, TRAF6, TRAF2, and MALT1 [21,23].

We previously reported that RTA induces the degradation of vFLIP early in lytic reactivation, resulting in the termination of NF-κB signalling, presumably to promote transition from latency to lytic replication [24]. RTA-induced degradation of vFLIP is dependent on the activity of the Itch ubiquitin ligase [25]. We identified mutants of vFLIP that are unable to interact with Itch and cannot activate NF-κB [25]. Here we report that vFLIP interacts with the Itch and A20 and this interaction occurs independently of RTA. We propose that vFLIP inhibits A20 DUB activity to modulate NF-κB signalling through interference with negative regulation. We demonstrate reduced A20 activity and increased levels of RIPK1 ubiquitin conjugates following stimulation with TNFA in the presence of vFLIP. These observations support a model for how vFLIP counteracts the DUB activity of A20, allowing the virus to maintain latency.

## Results and discussion

### vFLIP interacts with Itch and A20

**Fig. 1.**
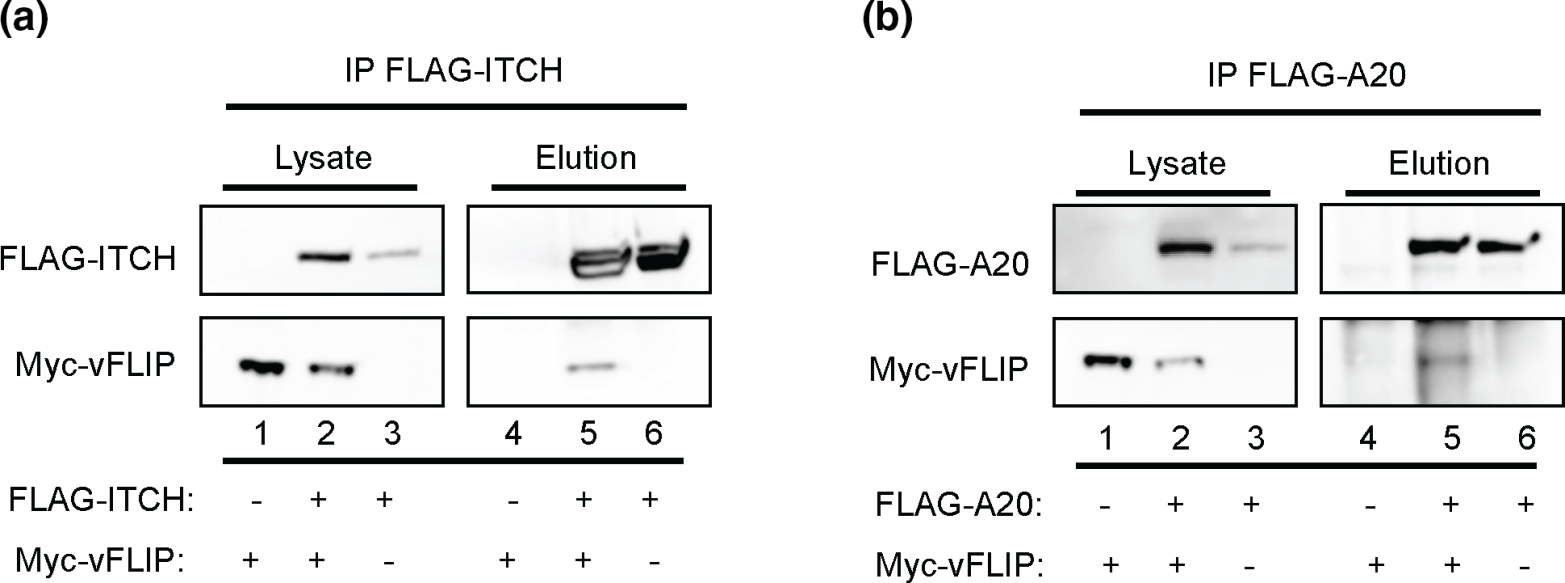
vFLIP interacts with Itch and A20. (a) vFLIP interacts with Itch. (b) vFLIP interacts with A20. For both experiments 293 T cells were transfected with Flag–Itch, Flag–A20, and/or myc–vFLIP, where indicated. Empty vector was used to maintain equal amounts of DNA. Itch or A20 were immunoprecipitated with Flag antibody-conjugated agarose beads and immunoprecipitates were analysed by immunoblot against Flag and myc. Data shown are representative of a minimum of three replicates.

We previously reported that RTA induces the degradation of vFLIP via the cellular ubiquitin ligase, Itch [25]. We originally hypothesized that RTA was recruiting Itch to vFLIP to promote ubiquitination and degradation of the viral protein. Upon further characterization of the interactions between vFLIP and Itch as part of the Itch/A20 ubiquitin editing complex, we observed interactions between vFLIP and Itch and vFLIP and A20, in the absence of RTA (Fig. 1a, b). Based on these observations, we hypothesized that vFLIP interacts with Itch and A20, either as a complex or binary interaction, to modulate A20 activity.

### vFLIP inhibits the deubiquitinase activity of A20

**Fig. 2.**
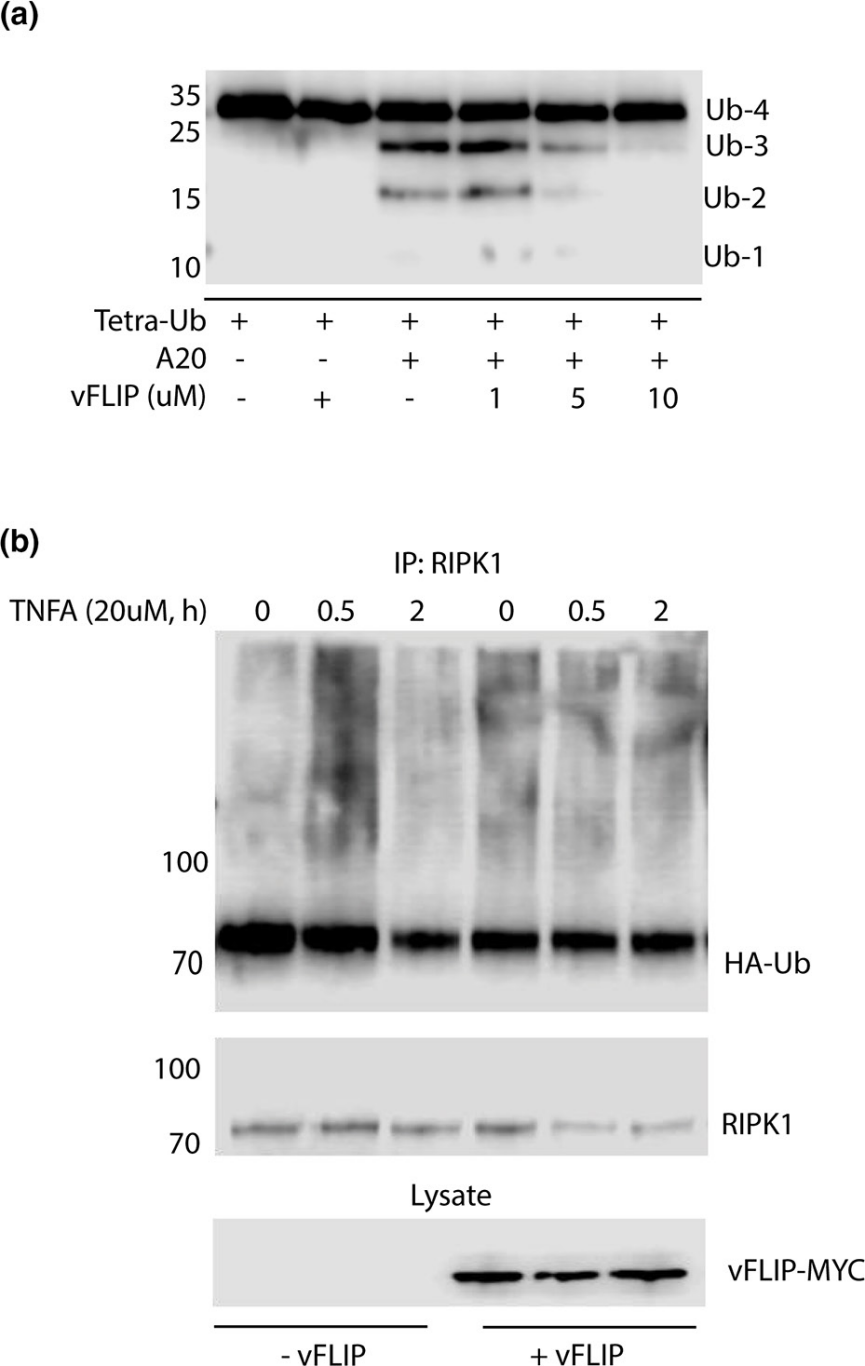
vFLIP modulates A20 DUB activity. (a) vFLIP inhibits A20 DUB activity *in vitro*. In 20 µl reactions the following reagents were added where indicated: tetra-K63 Ub (Boston Biochem) (500 nM), V5-vFLIP (1,5,10 µM), and A20 (2 µM). Reactions were incubated at 37 °C for 2 h following addition of 4× Laemmli loading buffer. Samples were run on 15 % SDS-PAGE gel and analysed via immunoblot with antibody against ubiquitin. (b) vFLIP abrogates the deubiquitination of RIPK1. HEK-293T cells were transfected with either empty vector control, HA-tagged WT Ub, and myc-tagged vFLIP where indicated, and 24 h post-transfection cells were treated with TNFA at 0, 0.5, and 2 h before harvesting, and analysed via immunoprecipitation with anti-RIPK1. Following immunoprecipitation, lysates were analysed by immunoblotting with anti-HA and anti-RIPK1. Data shown are representative of a minimum of three replicates.

A20 is a well-characterized negative regulator of NF-κB signalling. Following stimulation of NF-κB via the TNF receptor (TNFR), A20 downregulates signalling by removal of K63-linked polyubiquitin chains from RIPK1 and, in concert with Itch, adds K48 linked polyubiquitin, resulting in RIPK1 degradation via the proteasome. It was previously reported that vFLIP induces the expression of A20. It has been proposed that A20 expression, in the context of latent KSHV infection, is necessary to limit the inflammatory phenotype induced by persistent NF-κB signalling. We hypothesized that A20 activity needs to be tightly regulated, as excessive activity has the potential interfere with latency and cell survival, and inhibition of NF-κB signalling has been shown to promote apoptosis and lytic reactivation. To this end, we assessed the impact of vFLIP on A20 DUB activity. Using purified K63-linked tetraubiquitin, A20, and vFLIP, we evaluated A20 DUB activity via *in vitro* assay. Addition of purified A20 alone to tetraubiquitin resulted in cleavage of tetraubiquitin to faster migrating mono- and polyubiquitin species (Ub-3, Ub-2, Ub-1); however, addition of recombinant vFLIP resulted in a dose-dependent decrease in DUB activity (Fig. 2a).

A well-characterized target of A20 DUB activity is RIPK1, following TNFR stimulation. Within 30 min of TNFR stimulation, transient K63-polyubiquitin conjugates of RIPK1 can be detected via Western blot. By 2 h post-stimulation, K63 ubiquitin conjugates are removed by A20. To determine whether vFLIP inhibits DUB activity in the context of NF-κB signalling, we evaluated RIPK1 ubiquitin conjugates following stimulation with 20 µM TNFA. Cells were transfected with HA-tagged ubiquitin and vFLIP where indicated. Endogenous RIPK1 was purified and immunoprecipitates were probed for HA-tagged ubiquitin. Control cells, lacking vFLIP, displayed the characteristic increase in RIPK1 ubiquitin conjugates after 30 min of TNFA treatment, followed by deubiquitination 2 h post-treatment (Fig. 2b). vFLIP expression, however, resulted in detection of sustained RIPK1 ubiquitin conjugates regardless of TNFA stimulation beginning at *t*=0 (Fig. 2b). These data, taken together, suggest that vFLIP may modulate A20 DUB activity.

### Discussion

We have presented multiple observations supporting a novel mechanism by which vFLIP may promote NF-κB signalling and maintain latency. vFLIP is an established activator of NF-κB signalling and this activity is associated with viral latency. However, activation of NF-κB results in the expression of several negative regulators of the signalling pathway. Expression of one such negative regulator, A20, was shown to be induced by vFLIP. While NF-κB signalling is important for maintaining latency, prolonged NF-κB activation could contribute to an inflammatory phenotype. In fact, this is what occurs when negative regulators of NF-κB are either naturally or experimentally defective. Deficiencies in Itch ubiquitin ligase expression or function are associated with immune deficiencies and the Itch −/− knock out mouse displays an ‘itchy’ phenotype for which this gene is named [26]. A20 −/− mice also display a phenotype associated with inflammation and autoimmunity, exhibiting hypersensitivity to TNF and premature death [27]. To establish and maintain a latent infection, vFLIP must activate NF-κB and signalling must be sustained without killing the host, and, to accomplish this, the virus must control negative regulators of NF-κB.

Here we report an observed interaction of vFLIP with Itch and A20 either as complex or through binary interactions. We previously reported that in the presence of RTA, Itch targets vFLIP for degradation. These recent observations suggest that vFLIP may be interacting with Itch and A20 in latency and reactivation and may be modulating the activity of this complex. We reasoned that vFLIP interaction with the Itch/A20 ubiquitin editing complex may function to promote NF-κB signalling, and expression of RTA abrogates signalling by inducing the degradation of vFLIP as well as other members of the complex.

We observed, through *in vitro* assay and through immunoprecipitation of RIPK1 conjugates, inhibition of A20 DUB activity by vFLIP. Detection of sustained ubiquitinated RIPK1 in the presence of vFLIP suggests that A20 DUB activity is limited, thereby allowing for constitutive NF-κB signalling. The *in vitro* deubiquitination assay was carried out using A20 purified from 293 T cells under denaturing conditions to reduce co-purified proteins, although it is possible that Itch was copurified with A20, so a role for Itch in the observed inhibition of A20 DUB activity cannot be ruled out.

Taken together, we provide evidence for interaction of vFLIP with A20 and Itch; however, it is unclear whether vFLIP is interacting with this ubiquitin-editing complex, individual ubiquitin-editing proteins, or complex containing additional yet-to-be-identified components. We provide evidence for the functionality of this interaction through *in vitro* deubiquitination of tetraubiquitin and sustained ubiquitination of RIPK1. Additional work needs to be done to further characterize the nature of the interaction between vFLIP, Itch and A20 and determine the impact on viral latency and lytic reactivation.

## Methods

### Cell line maintenance and transfection

Human embryonic kidney 293T (HEK-293T) cells were cultured in DMEM supplemented with 10 % foetal bovine serum and were grown in 5 % CO_2_ at 37 °C. Cells were transfected at 60–70 % confluency using 1 µg ml^−1^ polyethyleneimine (PEI) linear, MW ~25 000 (Polysciences, Inc., cat# 23966) at a ratio of 1 µg plasmid DNA : 3 µl PEI. After 5 min of incubation the mixture was added to the cells. For the RIPK1 IP, 24 h post-transfection the media was changed and 2.5 µM of MG132 was added.

### Reagents, plasmids, and antibodies

The proteasome inhibitor MG132 (Boston Biochem) was used in this study. Flag–A20 was provided by Ed Harhaj, Flag–Itch was provided by Annie Angers [28]and myc–vFLIP by Gary Hayward. The following primary antibodies were used: anti-cMyc (Millipore), anti-M2 Flag (Sigma-Aldrich) and anti-A20 (BD Transduction Laboratories). The secondary antibodies used were anti-mouse-HRP and anti-rabbit-HRP (Jackson ImmunoResearch).

### Immunoblot analysis

Proteins were run on 12 % Tris–glycine or Any kD mini-PROTEAN Precast Gel (Biorad) with Tris–glycine running buffer. The proteins were then transferred to a PVDF membrane using a semi-dry transfer system at 20V for 20 min. The membranes were blocked in 5 % non-fat dry milk in PBS for 1 h. Primary antibodies were prepared in with 2.5 % non-fat dry milk at 1 : 1000 dilutions and applied to the membranes. The membranes were incubated on a shaker at 4 °C overnight and were washed in PBS containing 0.1 % Tween the following day. Secondary antibodies were prepared in 2.5 % non-fat dry milk at 1 : 1000 dilutions and applied to the membranes. The membranes were incubated at room temperature on a shaker for 1 h and afterward were washed with PBS containing 0.1 % Tween. Proteins were visualized with the addition of ECL substrate and the detection of the luminescence on X-ray film or scanned by a Li-COR C-DiGit Blot Scanner.

### Immunoprecipitation

Immunoprecipitations were carried out using the Flag immunoprecipitation kit (MilliporeSigma). Approximately 1.76×10^7^ cells transfected with the indicated constructs were harvested 48 h post-transfection by incubation with 1 ml of lysis buffer supplemented with protease inhibitor and N-ethylmaleimide. Cell lysates were centrifuged at 10 000 r.p.m. for 5 min to remove cell debris. The resulting supernatant was incubated with prepared Flag agarose beads for 3 h and washed 4× with wash buffer. Protein complexes were eluted with 100 µl of 150 ng µl^−1^ Flag peptide by rocking for 30 min at 4 °C. Immunoprecipitates were visualized through immunoblot analysis as described above

### A20 purification

A20-Flag was transfected into 293 T cells and purified following 48 h incubation. Nine 10 cm dishes of A20 transfected cells were harvested in ice-cold PBS, centrifuged, and lysed in 1 ml lysis buffer [50 mM HEPES, pH 7.4, 100 mM KAc, 5 mM MgAc_2_, 100 µg ml^−1^ digitonin, 1 mM DTT, 1X EDTA-free Complete protease inhibitor cocktail (Thermo Scientific)] for 20 min on ice. The lysate was centrifuged to remove cell debris. Supernatant was incubated with 100 µl of anti-Flag M2 affinity resin (Sigma) for 1 h at 4 °C. The anti-Flag M2 affinity resin was washed three times in 1 ml lysis buffer, three times in 1 ml wash buffer A (50 mM HEPES, pH 7.4, 400 mM KAc, 5 mM MgAc_2_, 100 µg ml^−1^ digitonin, 1 mM DTT), and three times in wash buffer B (50 mM HEPES,pH 7.4, 100 mM KAc, 5 mM MgAc_2_). Purified protein was eluted 2× with one volume of 0.2 mg ml^−1^ 3X-Flag peptide in wash buffer B at room temperature for 30 min.

### *In vitro* deubiquitination assay

V5-His-tagged vFLIP was expressed in *E. coli* (BL21) and purified using Ni–NTA resin (Thermo Fisher). A20–Flag was purified as previously described. Purified tetra-K63 ubiquitin was purchased from Boston Biochem. The following reagents were added to 20 µl reactions where indicated: A20 (2 µM), vFLIP (1, 5, 10 µM), tetra-K63 Ub (500 nM). Reactions were incubated at 37 °C for 2 h followed by the addition of 4× Laemmli loading buffer. Reactions were analysed by SDS PAGE followed by immunoblot.

### Rigour and reproducibility

All experiments are repeated a minimum of three times. Representative experiments are shown.

## Abbreviations

A20: tumor necrosis factor interacting protein 3
DUB: deubiquitinase
KSHV: Kaposi's sarcoma herpesvirus
MCD: multicentric Castlemans disease
NF-κB: nuclear factor kappa b
PEL: primary effusion lymphoma
TNFA: tumor necrosis factor alpha
TNFR: tumor necrosis factor receptor
Ub: ubiquitin
vFLIP: viral FLICE inhibitory protein
